# Cytomegalovirus infection in critically ill patients: a systematic review

**DOI:** 10.1186/cc7875

**Published:** 2009-05-14

**Authors:** Ryosuke Osawa, Nina Singh

**Affiliations:** 1 Infectious Diseases Section, VA Medical Center, University Drive C, Pittsburgh, PA 15420 USA; 2 Division of Infectious Diseases, Department of Medicine, University of Pittsburgh 3601 Fifth Avenue, Falk Medical Building Suite 3A, Pittsburgh, PA 15213 USA

## Abstract

**Introduction:**

The precise role of cytomegalovirus (CMV) infection in contributing to outcomes in critically ill immunocompetent patients has not been fully defined.

**Methods:**

Studies in which critically ill immunocompetent adults were monitored for CMV infection in the intensive care unit (ICU) were reviewed.

**Results:**

CMV infection occurs in 0 to 36% of critically ill patients, mostly between 4 and 12 days after ICU admission. Potential risk factors for CMV infection include sepsis, requirement of mechanical ventilation, and transfusions. Prolonged mechanical ventilation (21 to 39 days vs. 13 to 24 days) and duration of ICU stay (33 to 69 days vs. 22 to 48 days) correlated significantly with a higher risk of CMV infection. Mortality rates in patients with CMV infection were higher in some but not all studies. Whether CMV produces febrile syndrome or end-organ disease directly in these patients is not known.

**Conclusions:**

CMV infection frequently occurs in critically ill immunocompetent patients and may be associated with poor outcomes. Further studies are warranted to identify subsets of patients who are likely to develop CMV infection and to determine the impact of antiviral agents on clinically meaningful outcomes in these patients.

## Introduction

Cytomegalovirus (CMV) is a major β herpes virus and a significant human pathogen. Infection is common with seroprevalence rates increasing steadily from 65% among 40 to 49 year olds to 91% in those aged 80 years or over [1]. After primary infection, CMV, like other β herpes viruses, establishes life-long latency. In immunocompetent individuals, asymptomatic viral shedding may be detectable in saliva or urine; however, cell-mediated host immune responses prevent the development of overt CMV disease.

In contrast, CMV infection has been shown to lead to significant disease in immunocompromised hosts such as those with HIV infection or transplant recipients. End-stage HIV-infected patients with a CD4 lymphocyte count of less than 50 cells/mm^3 ^are at the highest risk of developing CMV retinitis [2]. In transplant recipients, CMV disease occurs in 11 to 72% of patients especially in the first three months after transplant while the patients are receiving maximum immunosuppression [3]. In addition to febrile syndrome and end-organ disease directly as a result of viral replication, immunomodulatory characteristics of CMV may contribute to opportunistic infections, allograft rejection, and higher mortality in transplant recipients. Clinical trials have shown that preventive approaches utilizing antiviral agents have lead to a reduction in the rates of CMV infection and disease, and indirect sequelae associated with CMV [3-5]. Currently, prophylaxis or periodic monitoring and antiviral therapy targeted towards patients with viral replication are routinely employed at many transplant centers.

It has increasingly come to be recognized that critically ill patients who are traditionally considered immunocompetent may also be at risk for CMV infection. For example, septic insult as a result of bacterial or fungal infections has the potential to promote the release of immunomodulatory cytokines and lead to reactivation of CMV [6,7]. Reactivation from the latency rather than primary infection is believed to be the cause of CMV infection because none of the critically ill CMV seronegative patients developed CMV infection as opposed to 13 to 56% of seropositive patients [8,9]. Several observational studies have shown an association between CMV infection in critically ill patients and poor clinical outcomes [8,10,11]. However, available data are limited by relatively small sample sizes, diversity in patient populations studied, difference in methodological assays employed for CMV, and variability in reported outcomes that preclude generalizability of the results of the individual reports. The objectives of this review are to summarize the frequency and predictors of CMV infection, and outcomes in critically ill immunocompetent patients with CMV infection. Additionally, we discuss the pathophysiologic basis of CMV reactivation and the implications of these data for optimizing outcomes in critically ill patients.

## Materials and methods

English-language reports of published studies on CMV infection in critically ill immunocompetent patients were identified through November 2008 by cross-referencing the following medical subject headings (MeSH) keywords and text words: cytomegalovirus, immunocompetence, critical illness, intensive care units, intensive care, reactivation, sepsis, and shock. Databases searched included PubMed, EMBASE, Cochrane Database of Systematic Reviews, and Cochrane Central Register of Controlled Trials. Bibliographies of original articles were manually reviewed for additional articles. Non-English-language reports were also identified in PubMed using the same keywords in order to supplement our search.

We included studies in which: critically ill immunocompetent adults were monitored either retrospectively or prospectively for the development of CMV infection in the ICU and; the rate of CMV infection was explicitly reported. CMV infection was defined as evidence of positive viral cultures, antigenemia, and/or DNAemia by PCR from blood or a clinical specimen. Patients were considered to be immunocompetent if they were not solid organ or hematopoietic stem cell transplant recipients, not infected with HIV, did not have primary immunodeficiencies, and were not recipients of immunosuppressive agents such as calcineurin-inhibitors, anti-TNF-α drugs, anti-lymphocyte antibodies, or chemotherapeutic agents for treating cancer. We excluded studies in which an increase in CMV serologic titers in the absence of viremia was the sole evidence for CMV infection.

Two of the authors independently searched articles and extracted the following data for analyses: study design, inclusion criteria, type and frequency of CMV assays, rate of CMV infection, rate of CMV IgG positivity, the time elapsed from ICU admission to CMV infection, risk factors for CMV infection, and outcomes (i.e. mortality, duration of ICU stay). Any discrepancies were resolved by review and discussion. Authors of published studies were contacted if reported data required further clarification. Additional mortality data was provided in one study [12].

## Results

### Study characteristics

The initial database search identified 524 English-language and 77 non-English-language studies. After review of the title and abstract and manual search for bibliographies of the potentially relevant articles, 26 studies were selected for full-text review [6-31]. Ten studies were excluded after full-text review because: CMV infection was diagnosed based on an increase in titers [19-24]; the study was not explicitly conducted in the ICU [25]; or the rate of CMV infection was not reported [26,27]. Data from one institution with overlapping study cohorts in two articles were analyzed only once to avoid duplication in the results [16,28]. We found three studies in which CMV disease was sought as etiology of acute respiratory distress syndrome (ARDS) or ventilator-associated pneumonia (VAP) [29-31]. Considering that CMV disease (organ-specific symptoms or signs plus the detection of CMV in organ biopsy samples by histopathology) is a distinct entity with worse outcomes than CMV infection, the data from these studies were summarized separately.

Thus, we identified a total of 13 studies that have described CMV infection in immunocompetent critically ill patients with sample sizes ranging from 23 to 237 (Table 1) [6-18]. These included nine prospective observational studies [7,8,10-16], three retrospective studies [9,17,18], and one study where insufficient details were available to determine the type of the study [6]. CMV IgG was positive in all the subjects in four studies [10,11,14,16], not measured in four studies [12,15,17,18], and positive in 28 to 94% of the subjects in five studies [6-9,13]. Other inclusion criteria were sepsis in four studies [6,7,16,17], mediastinitis in one study [13], simplified acute physiology score (SAPS) II of 41 or higher in one study [10], prolonged ICU stay in three studies [8,9,16], fever for more than 72 hours in one study [18], and shock or organ failure in two studies [12,16]. There were five studies in which corticosteroid use was documented [8,12,16-18]. In one study, eight of 48 patients were recipients of long-term corticosteroid therapy or had a malignancy [12]. In a case-control study, 22 of 40 of the cases and 13 of 40 of the controls were recipients of short-term corticosteroids (≤ 3 months) [18]. The details of corticosteroid use were unavailable in three studies [8,16,17].

**Table 1:** Characteristics of studies assessing the rate and outcome of cytomegalovirus infection in critically ill patients

				CMV infection				Outcome CMV positive vs. negative	
				
Study	Inclusion criteria	Number of patients	CMV IgG^a^	Frequency of monitoring	Assay (specimen)	Rate^a^	Time to positivity, day^b^	Mortality, death/total (%)	ICU stay, day^b^
[13]	ICU, mediastinitis after cardiac surgery	115	22/78 (28)	Every 3 weeks	Culture (blood, urine)	29/115 (25)	37 ± 22^c^	16/29 (55) vs. 31/86 (36) (NS)	69 ± 36 vs. 48 ± 27 (*P *< 0.05)
					Culture (blood)	23/115 (20)			
[6]	ICU, sepsis	60	33/44 (75)	Once	Culture, antigenemia, PCR (blood)	43/44 (98)	ND	ND	ND
[14]	ICU, mechanical ventilation	23	23/23 (100)	Random^d^	PCR, culture (blood)	0/23 (0)	NA	NA	NA
				Random^e^	PCR, culture (BAL)	0/14 (0)			
[17]^f^	SICU, postoperative sepsis with no identifiable sources	142	ND	Once, day 30 ± 5^g^	Culture (blood, sputum, BAL)	12/142 (8.5)	NA	8/12 (66) vs. 45/130 (35)	ND
[7]	ICU, sepsis	34	31/33 (94)	Twice weekly	PCR (blood)	11/34 (32)	4 (1–23)	7/11 (64) vs. 17/23 (74) (NS)	ND
					Antigenemia	6/34 (18)	11 (1–23)		
[12]	ICU, ≥ 2 organ failures	48	ND	Once, day 1.8 ± 2.2^b^	PCR (blood)	1/48 (2.1)	NA	1/1 (100) vs. 15/47 (32) (NS)	ND
					Antigenemia	0/48 (0)			
[10]	ICU, SAPS II ≥ 41	56	56/56 (100)	Every week	PCR (blood, LRT secretions)	20/56 (36)	11	11/20 (55) vs. 13/36 (36) (NS)	30 vs. 23 (*P *= 0.0375)
					PCR (blood)	18/56 (32)			
					Culture (LRT secretions)	7/56 (13)			
					Culture (blood)	0/56 (0)			
[15]	ICU	120	ND	Once, day 4	PCR (blood)	1/120 (0.8)	NA	ND	ND
[8]	SICU stay ≥ 5 days	104	76/104 (73)	Every week	Culture (blood, LRT secretions)	10/104 (10)	28 ± 4^g^	5/10 (50) vs. 25/94 (27) (NS)	41 vs. 19 (*P *= 0.001)
					Culture (blood)	6/104 (5.8)			
[18]	ICU, fever > 72 hours without evidence of bacteriologic or fungal origin	237	ND	Clinical judgment	Antigenemia	40/237 (17)	20 ± 12	20/40 (50) vs. 11/40 (28) (*P *= 0.02)	41 ± 28 vs. 31 ± 22 (*P *= 0.04)
[16]	ICU stay ≥ 7 days, septic shock	25	25/25 (100)	Twice weekly in week 1, then every week	Antigenemia	8/25 (32)	7 (0–14)	5/8 (63) vs. 6/17 (35) (NS)	42 (16–87) vs. 18 (10–42) (*P *= 0.0025)
					Culture (blood, LRT secretions, urine)	4/25 (16)			
					Culture (blood)	1/25 (4)			
[11]	ICU^h^	120	120/120 (100)	Three times weekly	PCR (blood)	39/120 (33)	12 (3–57)	CMV viremia at any level is associated with continued ICU hospitalization or death at day 30 (OR 5.7, 95% CI 2.1–15.6)	
[9]	ICU stay ≥ 14 days	99	41/56 (73)	Random^i^	PCR (blood)	35/99 (35)^j^	17 ± 15	10/35 (29) vs. 7/64 (11) (*P *= 0.048)	33 ± 19 vs. 22 ± 11 (*P *< 0.001)

BAL = bronchoalveolar lavage; CI = confidence interval; CMV = cytomegalovirus; ICU = intensive care unit; LRT = lower respiratory tract; NA = not applicable; ND = no data available; NS = not significant; OR = odds ratio; PCR = polymerase chain reaction; SAPS = simplified acute physiologic score; SICU = surgical intensive care unit.

^a ^Data were presented as positive/total (%).

^b ^Data were presented as mean ± standard deviation or median (range) unless otherwise indicated.

^c ^Time was measured after heart surgery.

^d ^Blood was collected at a mean of five days for the first sample and 17 days (range: 6 to 29 days) for the others.

^e ^BAL was performed at a mean of 19 days (range: 1 to 41 days).

^f ^Data were presented as combined outcomes of patients with CMV or herpes simplex virus infection in the original article. Data regarding CMV infection were extracted for this review.

^g ^Data were presented as mean ± standard error of mean.

^h ^This included admission to the burn ICU with at least 40% body surface burn or at least 20% body surface burn with inhalation injury, to the trauma ICU with in Injury Severity Score > 15 and > 4 unit packed red blood cells within 24 hours, to the medical ICU with suspected or documented sepsis, or to the cardiac care ICU with a diagnosis of acute myocardial infarction.

^i ^Blood was collected randomly and was stored for red blood cell cross-matching. The first blood samples were drawn on day 6.6 ± 8.2, and the last blood samples were drawn on day 20 ± 16.

^j ^23 of 41 (56%) CMV seropositive patients developed CMV infection.

### Rate of CMV infection

Based on nine prospective studies that assessed CMV infection in all study subjects, the rate of CMV infection ranged from 0 to 36% with the median rate of 25% [7,8,10-16]. The specimens used to assess CMV infection included blood, urine, and respiratory secretions. All the studies used blood to assess CMV infection. Blood was used solely in four studies [7,11,12,15], whereas urine and respiratory specimen in addition to blood samples were used in two [13,16] and four studies [8,10,14,16], respectively. The rate of CMV infection was reported separately based on the type of specimens in these studies (Table 1). Considering only studies that assessed for viremia, the CMV infection rate ranged from 0 to 33% with the median rate of 20%. Thereafter, we reported the rate of CMV infection diagnosed based on the presence of viremia.

Next, studies were categorized based on the frequency of virologic monitoring because it can influence the rate of CMV infection. In five studies in which CMV infection was monitored at least weekly, the rate of CMV infection ranged from 6 to 33% with the median of 32% [7,8,10,11,16]. In contrast, CMV infection rate was 0.8 to 2.1% in two studies in which assessment for CMV infection was performed only once, 1.8 to 4 days after ICU admission [12,15].

There are three methods of diagnosing CMV infection: viral cultures, antigenemia, and PCR assays [32]. Culture-based assays (conventional and shell-vial cultures) are considered obsolete because of their low sensitivity and time-consuming nature. The antigenemia assay is based on direct detection of the CMV protein pp65 using monoclonal antibodies. It is sensitive and quantitative, although it requires sufficient leukocytes in peripheral blood and is more labor-intensive than the PCR assays. Finally, the PCR assays have been considered gold standard given their high sensitivity and rapid turnover time, although these are not fully standardized [33]. In our review, the assays used to assess CMV infection were viral cultures in five studies [8,10,13,14,16], antigenemia in three studies [7,12,16], and PCR in six studies [7,10-12,14,15]. CMV infection rate was 0 to 20% (median 4%), 0 to 32% (median 18%), and 0 to 33% (median 17%) by viral cultures, antigenemia, and PCR, respectively in these indices. PCR assay and antigenemia were performed in all patients in two studies [7,12]. Twelve of 82 patients developed CMV infection detected by the PCR assay compared with six of the 82 patients by antigenemia. No cases of CMV infection were diagnosed solely by antigenemia. Lastly, we focused on three studies in which the PCR assay was used at least weekly because this method is currently widely utilized to monitor CMV infection; the rate of CMV infection was 32 to 33% in these studies [7,10,11].

### Time to onset of CMV infection

Based on five studies where CMV infection was assessed at least weekly, the mean (or median) time to onset of CMV infection ranged from 4 to 28 days [7,8,10,11,16]. Among studies where PCR was used, the mean (or median) time ranged from 4 to 12 days [7,10,11]. A PCR assay seems to help diagnose CMV infection earlier than the antigenemia assay. In a study that used both methods for the diagnosis of CMV infection, the median time between onset of sepsis and CMV infection determined by PCR and antigenemia were four days (range 1 to 23 days) and 11 days (range 1 to 23 days), respectively [7].

### Risk factors for CMV infection

Candidate variables assessed as predictors for CMV infection varied for different studies and primarily included demographic and clinical characteristics, and severity of illness markers. Age did not appear to be a risk factor for CMV infection [7,8,10,11,13,16,18] and the association between CMV infection and gender was inconsistent [7,8,10,11,13,16,18]. Other risk factors identified included mechanical ventilation at admission (odds ratio [OR] 8.5, 95% confidence interval [CI] 1.1 to 66.5 for high-grade CMV viremia, i.e. CMV PCR > 1000 copies/ml) [11], bacterial pneumonia [8], and sepsis (OR 4.62, *P *= 0.02) [10]. Corticosteroid use was a risk factor in one study [8]. In a retrospective case-control study where variables used in a multivariate logistic regression model were not clearly documented, neither corticosteroid use nor sepsis was a risk factor for CMV infection [18]. Transfusion within 24 hours of admission was associated with high-grade viremia, i.e. CMV viral load greater than 1000 copies/ml (OR 6.7, 95% CI 1.1 to 42.7) [11]; however, no association between CMV infection and transfusion during hospitalization was documented in two other studies [18]. The mean number of packed red blood cell transfusion was larger in patients with CMV infection than in those without it (22.3 units vs. 11.2 units, *P *= 0.002); however, this difference was not statistically significant after controlling for other risk factors [8]. Malignancy was not associated with CMV infection [9,10,18]. None of the disease severity scores including Acute Physiology and Chronic Health Evaluation (APACHE) II score [7,8,11,13], sepsis-related organ failure assessment (SOFA) score [16], or SAPS II [10,18] correlated with a risk of CMV infection.

### Effect of CMV infection on outcomes

#### Organ dysfunction

Organ dysfunction was reported in three studies [8,13,18]. One study showed a higher incidence of hepatic dysfunction (international normalized ratio > 1.5 or total bilirubin > 2.5 mg/dL) in CMV infection group (70% vs. 36%, *P *< 0.047) [8]. In two studies, renal failure was observed more frequently in those with CMV infection (55 to 58% vs. 32 to 33%, *P *< 0.05 in each study) [13,18].

#### ICU stay

Based on six studies where the duration of ICU stay was assessed, the mean (or median) duration of ICU stay ranged from 33 to 69 days in patients with CMV infection as compared with 22 to 48 days among those without (*P *< 0.05 in each study) [8-10,13,16,18].

#### Mechanical ventilation

The mean (or median) duration of mechanical ventilation ranged from 21 to 39 days in patients with CMV infection compared with 13 to 24 days in those without CMV infection in four studies (*P *< 0.05 in each study) [8,9,16,18].

#### Bacterial or fungal infection

Nosocomial infection was more frequently observed in patients with CMV infection as compared with those without CMV infection in one study (75% vs. 50%, *P *= 0.04) [18].

#### Viral load and outcomes

A higher level of CMV viremia was associated with death or continued ICU hospitalization at 30 days in one study [11]. An OR of combined outcome of death or continued ICU hospitalization at 30 days was 1.7 (95% CI 1.2 to 2.4) for each logarithmic increase in maximum CMV viral load measured [11].

#### Mortality

Mortality rate in critically ill patients with CMV infection was 29 to 100% as compared with 11 to 74% in those without CMV infection. Except for two retrospective studies [9,18], no other studies showed a significant difference in the mortality rates between those with and without CMV infection. CMV viremia at any level was associated with death or continued ICU hospitalization at 30 days (OR 5.7, 95% CI 2.1 to 15.6) [11].

### Infections due to other herpes viruses

Human herpesvirus (HHV) -6 and -7 have been associated with a greater risk of developing CMV disease in transplant recipients [34-37]. Furthermore, HHV-6 and HHV-7 have been shown to be significant contributors to morbidity and poor outcomes, particularly when concurrent infection with CMV exists [35,38-40]. Thus, it is of interest to investigate whether the association between CMV and other herpes viruses exists in critically ill patients.

#### Human herpesviruses -6 and -7

HHV-6 infection has been frequently observed in critically ill patients [12,15]. In one study, HHV-6 infection occurred in 53.5% of all patients (54/101) requiring hospitalization in the ICU as compared with none of the healthy volunteers [15]. In another study, HHV-6 infection occurred in 54% of all ICU patients with at least two organ failures as opposed to 15% of those with less than two organ failures [12]. HHV-6 viremia was assessed by the PCR assay at a mean of day 4 and day 1.8, respectively [12,15]. Potential association between CMV and HHV-6 infection could not be assessed because only one patient developed CMV infection in each study. Both studies failed to show an association between HHV-6 infection and mortality. HHV-7 infection was observed more commonly in healthy volunteers (18/50, 36%) as compared with ICU patients (14/101, 14%; *P *= 0.002) in one study [15].

#### Herpes simplex virus

Three studies evaluated herpes simplex virus (HSV) infection in conjunction with CMV infection. In one study, HSV was isolated in the bronchial aspirate of 8 of 25 patients (32%) consisting of six of eight patients with CMV reactivation and 2 of 17 patients without CMV infection (*P *= 0.004) [16]. In other studies, none or only one patient developed co-infection of CMV and HSV, therefore, the association between CMV and HSV could not be evaluated [8,17].

### CMV disease and acute respiratory distress syndrome or ventilator-associated pneumonia

CMV pneumonia was diagnosed by open-lung biopsy while investigating the etiology of pulmonary disease in 29 to 50% of critically ill patients with ARDS or VAP (Table 2) [29-31]. In a study in which open-lung biopsy led to the diagnosis of CMV pneumonia in 30% of critically ill patients with ARDS, lung tissue culture was positive only in 10% of these patients [29]. The sensitivity/specificity of bronchoalveolar lavage (BAL), blood, and urine culture for the diagnosis of histologically proven ventilator-associated CMV pneumonia was 53%/92%, 20%/83%, and 13%/62%, respectively [31]. Clinical outcome data were largely lacking; however, there was no difference in duration of ICU stay in one study [31].

**Table 2:** Characteristics of studies assessing the rate of cytomegalovirus pneumonia in critically ill patients

				CMV infection			Outcome CMV positive vs. negative	
				
Study	Inclusion criteria	Number of patients	CMV IgG^a^	Time of histologic examination, day	Methods	Rate^a^	Mortality, death/total (%)	ICU stay
[31]	ICU, acute respiratory failure or VAP, negative BAL cultures	86	13/18 (72)	18 (10–40)^b^	Autopsy or open-lung biopsy	25/86 (29)	ND	No difference
[30]	ICU, ≥ 5 days of evolution of ARDS, negative microbiological cultures	36	ND	10 (5–55)^c^	Open-lung biopsy	18/36 (50)	ND	ND
[29]	ICU, ≥ 5 days of evolution of ARDS, negative microbiological cultures	100^d^	ND	7 (6–13.5)^c^	Open-lung biopsy	30/100 (30)	ND	ND
					Lung tissue culture	10/100 (10)		

ARDS = acute respiratory distress syndrome; BAL = bronchoalveolar lavage; CMV = cytomegalovirus; ICU = intensive care unit; NA = not applicable; ND = no data available; VAP = ventilator-associated pneumonia.

^a ^Data were presented as positive/total (%).

^b ^Time was measured after the ICU admission.

^c ^Time was measured after the onset of ARDS.

^d ^Four immunocompromised patients were included.

## Discussion

Our review demonstrates that depending on the methodological assay used and the patient populations studied, CMV infection occurs in 0 to 36% of the critically ill otherwise immunocompetent hosts in the ICU. Among the most frequently studied inciting event for CMV infection in these patients is sepsis [6,7,13,16,17]. The risk of CMV infection was five-fold higher in patients with sepsis even when controlled for age and the initial severity of illness [10]. In a murine model of CMV infection, cecal ligation and puncture resembling post-surgical intraabdominal sepsis led to reactivation of latent CMV in the lungs and ultimately pulmonary fibrosis [41,42]. The propensity of sepsis to promote CMV infection may result from its pleiotropic effects on the host immune system. Pro-inflammatory cytokine production such as TNF-α and IL-1β in the early phase of sepsis has the potential to activate NF-κB and other transcription factors that are key in the reactivation of CMV from latency [43,44]. The later phase of sepsis, characterized by the generation of immunosuppressive cytokines such as IL-10 and IL-4 is often referred to as compensatory anti-inflammatory response syndrome [45,46]. Once latent virus is reactivated, these cytokines may further enhance CMV replication. Indeed, in lung transplant recipients, elevated levels of IL-10 in the BAL and/or plasma were associated with delayed CMV clearance [47]. A sustained high level of IL-10 in patients with sepsis has been associated with poor outcomes, presumably due to excessive anti-inflammatory effects [48].

Transfusion within 24 hours of admission was identified as a risk factor for high-grade CMV viremia in critically ill patients [11]. This association may be explained by potential transmission of CMV by blood products, but more likely by the immunomodulatory effect of transfusion *per se*. Previous studies have shown that allogeneic blood transfusion resulted in a reduction in T-helper cells, induction of suppressor T cells, and suppression of natural killer cell activity [49]. The transfusion-related immunosuppression has been associated with clinically important sequelae such as improvement of renal allograft survival, increased risk of tumor recurrence, and postoperative infections [50-52]. The risk of CMV transmission by leukocyte depleted blood products is at least as low as by CMV seronegative blood products [53,54], supporting the hypothesis that transfusion-related immunomodulatory effect plays a major role in CMV infection in critically ill patients if transfusion were truly a risk factor. However, these data were not available for most studies. For example, only one in three studies that evaluated transfusion as potential risk factors for CMV infection reported use of leukocyte depleted blood products explicitly [18].

A body of literature based largely on serologic assays for the diagnosis of CMV suggests that severe burn injuries are a major risk factor for CMV infection [55,56]. At least a four-fold rise in serologic titers suggestive of CMV reactivation has been documented in 45 to 56% of the burn patients [19,21,22]. Recently, in a study where patients with severe burn injuries comprised a subset of critically ill patients, CMV viremia using PCR was observed in 55% (11/20) of the burn patients [11]. Burn injuries are associated with profound changes in cell-mediated immunity and a predominant T-helper 1 cell response that may facilitate CMV infection [57-59]. Susceptibility to sepsis due to the loss of skin integrity in these patients may also contribute to the risk of CMV infection.

Attempts to utilize the severity of 'critical illness' to predict CMV infection have not shown a correlation between scoring systems such as APACHE II or SAPS II and the risk of CMV [7,8,10,11,13,16,18]. Severity of illness scores have typically been assessed in the first 24 hours after ICU admission whereas CMV infection does not usually occur until late in the ICU stay. Additionally, these scores are based on age, physiologic parameters, basic laboratory values, and chronic medical conditions and may not be necessarily representative of host immunologic deficits that lead to CMV infection.

CMV infection rate was 0.8 to 2.1% in two studies where the PCR assay was performed only once at a mean of 1.8 and 4 days following the onset of illness requiring ICU admission [12,15]. The median time to first detectable CMV viremia was 12 days (range 3 to 57 days) in a study where the PCR assay was performed thrice weekly [11]. Thus, it appears that CMV infection is a rare event very early in the course of critically ill patients and that most infections develop between 4 and 12 days after the onset of illness requiring ICU stay, which could lead to a hypothesis that CMV infection may coincide with the development of compensatory anti-inflammatory response syndrome, and not with the initial surge of pro-inflammatory cytokines.

A key question is whether CMV infection adversely affects outcomes in critically ill patients. Virtually all studies have documented that CMV infection was related to prolonged mechanical ventilation and duration of ICU stay in patients with CMV infection. CMV infection has also been associated with organ system failure and at least two studies have documented significantly higher mortality rates in patients with CMV infection compared with those without it [9,18]. Thus, although these data do not prove a causal association as CMV infection may have been more likely to develop or diagnosed in sicker patients, existing evidence suggests that CMV infection is associated with poor outcomes even in immunocompetent critically ill patients. We believe that a causal association between these can only be assessed by carefully conducted clinical trials designed to show whether suppression of CMV has a mitigating effect on the severity of illness.

Another major unresolved issue is whether CMV infection is associated with overt disease or clinical manifestations directly attributable to this virus in critically ill patients. CMV infection in immunocompetent patients generally presents with mononucleosis-like symptoms including fever and malaise with liver enzyme abnormalities [60,61], which are typically benign. However, 31 to 42% of the hospitalized patients with CMV infection may have organ involvement [60,62] and rarely life-threatening CMV infection has also been reported [63,64]. In critically ill patients, 10% (2/20) of those with CMV infection eventually developed severe CMV disease (pneumonitis, neurologic disease) in one study [10]. CMV pneumonia has also been diagnosed in 29 to 50% of patients with ARDS or VAP [29-31]; however, this does not necessarily mean that CMV is the cause of ARDS or VAP. Critical illness due to serious pulmonary disease may predispose these patients to CMV infection in the lungs. In a cohort study in the ICU, 17% of critically ill patients who experienced fever for three or more days had CMV infection [18]. Current guidelines for the evaluation of new fever in critically ill adult patients list transfusion-associated CMV mononucleosis as a cause of fever [65]. However, it remains to be determined whether and how often CMV produces febrile syndrome and whether coexistent infection with HHV-6 is a contributor to this entity as shown in the transplant setting [34,36-40].

Experimental studies have shown that ganciclovir prevented murine CMV reactivation and the development of pulmonary fibrosis in immunocompetent mice with sepsis [42]. Two retrospective studies where small subsets of ICU patients received antiviral agents for CMV infection have yielded inconclusive results and data on the utility and efficacy of antiviral therapy for CMV in critically ill immunocompetent patients are largely lacking [17,18]. Employment of potent antiviral therapy in all critically ill patients may be impractical, logistically infeasible, and potentially harmful given a large number of ICU patients and potential adverse effects of ganciclovir such as bone marrow suppression or teratogenicity. A more prudent approach may be to identify subgroups of patients at high risk for developing CMV infection and targeting antiviral prophylaxis towards these patients. These subgroups may include patients with sepsis, persistent fever, or those receiving transfusion. An alternative approach is to employ antiviral therapy only in those with CMV viremia. Regardless, carefully conducted clinical trials are warranted to discern the impact of antiviral agents on clinically meaningful outcomes before employing antiviral therapy or even considering routine monitoring of CMV in critically ill patients.

Several limitations of our study deserve to be acknowledged. We found considerable heterogeneity in the methodology used to assess CMV infection and in patient characteristics. As noted in the Results, the frequency and type of CMV monitoring influenced the rate of CMV infection. Although all the studies in this review were conducted in the ICU, the overall mortality of the studied patients ranged from 5 to 71% [7,15], suggesting that study populations were significantly diverse. Furthermore, these studies were published over a period spanning nearly two decades in different regions with diverse clinical practices. Considering the heterogeneity of available data, quantitative analyses such as meta-analysis can be misleading [66,67] and our results are therefore presented in a descriptive fashion only. Second, while we excluded the recipients of iatrogenic immunosuppressive agents that enhance the risk of CMV reactivation, critically ill patients in whom corticosteroids were employed were included. Controversy abounds whether corticosteroids alone without other immunosuppressive agents lead to reactivation of CMV from latency or merely promote the replication of activated virus [68-70]. Corticosteroids were employed in a subset of patients in 5 of 12 studies in this review. Given that corticosteroid use is a common practice in the ICU [71], these studies reflect clinical scenarios encountered by care providers and therefore their inclusion in this review was deemed appropriate.

## Conclusions

In summary, accumulating data suggest that CMV infection is a frequent occurrence in critically ill patients. Considering a large number of patients requiring ICU level of care, the scope of impact of CMV infection in these patients may be equally or potentially wider than in other immunocompromised hosts traditionally recognized to be at risk for CMV infection. For example in the USA, an estimated 383,000 cases with sepsis require ICU admission as opposed to 28,360 solid organ transplant cases yearly [72,73]. Mortality rate in patients with sepsis ranges from 20 to 50% and approaches 70% in those with multiple organ failure [74]. Furthermore, the incidence of sepsis and the number of sepsis-related deaths appear to be increasing [73,74]. Precise identification of the role of CMV as a contributor to outcomes in these patients may therefore have far reaching implications. Subsets of critically ill patients who are at risk for developing CMV infection and for poor outcomes remains to be determined. These studies have significant implications for future investigations to determine the potential benefits and for guiding the study design to evaluate the impact of antiviral agents on clinically meaningful outcomes in critically ill patients.

## Key messages

• CMV infection occurs in 0 to 36% (median 25%) of critically ill patients between 4 and 12 days after ICU admission, especially those with sepsis, requiring mechanical ventilation, and receiving transfusion.

• CMV infection is associated with poor outcomes; however, it is not known whether the causal association exists, that is, CMV is truly a pathogen or CMV infection is just an indicator of immunosuppression.

• It remains to be determined whether CMV produces febrile syndrome or end-organ disease directly in critically ill patients.

• Further studies are warranted to identify subsets of patients who are at high risk of developing CMV infection and to determine the role of antiviral agents on clinically important outcomes in critically ill patients.

## Abbreviations

APACHE: Acute Physiology and Chronic Health Evaluation; ARDS: acute respiratory distress syndrome; BAL: bronchoalveolar lavage; CI: confidence interval; CMV: cytomegalovirus; HHV: human herpesvirus; HSV: herpes simplex virus; ICU: intensive care unit; IL: interleukin; MeSH: medical subject headings; NF-κB: nuclear factor-κB; OR: odds ratio; PCR: polymerase chain reaction; SAPS: simplified acute physiology score; SOFA: sepsis-related organ failure assessment, TNF: tumor necrosis factor; VAP: ventilator-associated pneumonia.

## Competing interests

The authors declare that they have no competing interests.

## Authors' contributions

RO participated in the study design, literature search, data acquisition, interpretation of the data, and the drafting of the manuscript. NS participated in the study design, literature search, data acquisition, interpretation of the data, and the revision and editing of the manuscript. Both authors read and approved the final manuscript.
